# Phototherapy as a way to improve the psychological well‐being of older adults during the COVID‐19 pandemic

**DOI:** 10.1002/nop2.1590

**Published:** 2022-12-28

**Authors:** Alireza Davar, Mehrnoosh Partovirad, Hanieh Saveie, Nasrin Nikpeyma

**Affiliations:** ^1^ Saba Faculty of Arts and Architecture Shahid Bahonar University of Kerman Kerman Iran; ^2^ Department of Community Health and Geriatric Nursing, School of Nursing and Midwifery Tehran University of Medical Sciences Tehran Iran

The COVID‐19 pandemic has affected the psychological well‐being of older adults. It seems phototherapy, as a kind of art therapy, is a way to promote psychological well‐being in older adults by creating a deeper and more comprehensive meaning, reducing feelings of loneliness, depression and anxiety, and bringing happiness, love, intimacy, optimism and hope to them.

The COVID‐19 pandemic, one of the greatest global health crises, has had severe effects on various aspects of the physical, mental, social and emotional health of older adults (Lind et al., 2021).

The number of older adults suffering from grief, emotional stress, fear, diminished happiness, depression, loneliness and social isolation has been reported to increase dramatically due to social constraints and quarantine due to the COVID‐19 pandemic, and these conditions have affected the psychological well‐being of older adults (Chan et al., 2021; Holaday et al., 2022). Psychological well‐being means experiencing positive emotions and relationships with others and having a purposeful and meaningful life (Salehinejad et al., 2020). Psychological well‐being is known as a way to develop individual abilities and potential. Achieving psychological well‐being requires optimal physical, mental and social status (Ryff, 2013).

The results of studies show that diseases, crises and pandemics have been directly associated with a decrease in psychological well‐being in older adults (Olyani & Peyman, 2021). Several interventions such as art therapy have been performed to reduce the psychological consequences for older adults during the COVID‐19 pandemic (Albañil‐Delgado et al., 2020). There are several types of art therapy, and phototherapy is a way to promote psychological well‐being.

Phototherapy refers to any therapeutic use of photographs, and not necessarily to formal counselling and psychotherapy (Wheeler, 2015). In this definition, a photograph is used independently or as an auxiliary tool in art therapy. Phototherapy also refers to looking at a photograph and analysing it and is a process that begins when a photograph is taken, affects the mind of the audience each time they look at it, and ultimately leads to a therapeutic change (Wheeler, 2015). Phototherapy can provide the conditions for self‐awareness, improving family and social relationships, and increasing visual literacy skills, and can be considered an effective way to improve the mental health of vulnerable people (Glaw et al., 2017).

Stimulating, reinforcing and reflecting on positive thoughts and feelings recorded in photographs can give meaning to older people's lives and help them focus more on their values and emotional interactions, and this helps to minimize the psychological suffering caused by staying at home and social isolation. Also, photographs evoke the ups and downs of past lives and help older adults to hope for the future because difficulties and problems are not permanent (Lind et al., 2021; Testoni et al., 2022). Phototherapy displays thoughts and feelings that cannot be conveyed verbally. Looking at photographs helps older adults to remember the important people in their lives, the achievements and the bitter‐sweet experiences of the past, and to accept that life is still worth living despite its various challenges (Carretero et al., 2020). Looking at photographs of important life events (such as births, marriages and honours) or family members (children and grandchildren) by creating a deeper and more comprehensive meaning (Keisari et al., 2022), reduces feelings of loneliness, depression and anxiety in the older adults and brings happiness, love, intimacy, optimism, hope and psychological well‐being to them (Gantumur et al., 2020).

Therefore, due to ease of use, no need for high skills or expertise, cost‐effectiveness and the possibility of implementation in any place, phototherapy can be considered a therapeutic approach in crises and pandemics such as COVID‐19 to improve the psychological well‐being of older adults. One of the important roles of nurses is patient advocacy and attention to the physical, psychological, social and cultural needs of the client. Nurses can pay attention to the psychological needs of older adults by using phototherapy, a subcategory of art therapy, and it seems that it will have a positive effect on other aspects of the health of older adults.

## AUTHOR CONTRIBUTIONS

A.D was involved in study conception. M.P, H.S and N.N were involved in data collection. N.N was involved drafting and approving of the article.

## FUNDING INFORMATION

Not applicable.

## Data Availability

Not applicable.
